# RNA-Mediated Gene Silencing of Nicotinamide N-Methyltransferase Is Associated with Decreased Tumorigenicity in Human Oral Carcinoma Cells

**DOI:** 10.1371/journal.pone.0071272

**Published:** 2013-08-21

**Authors:** Valentina Pozzi, Davide Sartini, Stefano Morganti, Rachela Giuliante, Giulia Di Ruscio, Andrea Santarelli, Romina Rocchetti, Corrado Rubini, Marco Tomasetti, Giovanni Giannatempo, Fiorenza Orlando, Mauro Provinciali, Lorenzo Lo Muzio, Monica Emanuelli

**Affiliations:** 1 Department of Clinical Sciences, Polytechnic University of Marche, Ancona, Italy; 2 I.R.C.C.S. I.N.R.C.A., Ancona, Italy; 3 Department of Biomedical Sciences and Public Health, Polytechnic University of Marche, Ancona, Italy; 4 Department of Molecular and Clinical Sciences, Polytechnic University of Marche, Ancona, Italy; 5 Department of Clinical and Experimental Medicine, University of Foggia, Foggia, Italy; 6 Experimental Animal Models for Aging Units, IRCCS-INRCA, Ancona, Italy; 7 Advanced Technology Center for Aging Research, Scientific Technological Area, IRCCS-INRCA, Ancona, Italy; The University of Hong Kong, China

## Abstract

Oral squamous cell carcinoma (OSCC) is the most common type of oral cancer. Despite progress in the treatment of OSCC, overall survival has not improved substantially in the last three decades. Therefore, identification of reliable biomarkers becomes essential to develop effective anti-cancer therapy. In this study, we focused on the enzyme Nicotinamide N-methyltransferase (NNMT), which plays a fundamental role in the biotransformation of many xenobiotics. Although several tumors have been associated with abnormal NNMT expression, its role in cancer cell metabolism remains largely unknown. In this report, 7 human oral cancer cell lines were examined for NNMT expression by Real-Time PCR, Western blot and HPLC-based catalytic assay. Subsequently, we evaluated the *in vitro* effect of shRNA-mediated silencing of NNMT on cell proliferation. *In vivo* tumorigenicity of oral cancer cells with stable knockdown of NNMT was assayed by using xenograft models. High expression levels of NNMT were found in PE/CA PJ-15 cells, in keeping with the results of Western blot and catalytic activity assay. PE/CA PJ-15 cell line was stably transfected with shRNA plasmids against NNMT and analyzed by 3-(4,5-dimethylthiazol-2-yl)-2,5-diphenyl tetrazolium bromide (MTT) and soft agar Assays. Transfected and control cells were injected into athymic mice in order to evaluate the effect of NNMT silencing on tumor growth. NNMT downregulation resulted in decreased cell proliferation and colony formation ability on soft agar. In athymic mice, NNMT silencing induced a marked reduction in tumour volume. Our results show that the downregulation of NNMT expression in human oral carcinoma cells significantly inhibits cell growth *in vitro* and tumorigenicity *in vivo*. All these experimental data seem to suggest that NNMT plays a critical role in the proliferation and tumorigenic capacity of oral cancer cells, and its inhibition could represent a potential molecular approach to the treatment of oral carcinoma.

## Introduction

Oral squamous cell carcinoma (OSCC) accounts for about 90% of oral malignancies, and is the third most common cancer in developing nations and the sixth most prevalent tumor worldwide [1]. Treatment of OSCC is usually based on surgery or radiation, with or without concomitant chemotherapy. Despite the advances in surgical techniques, general patient care, and local and systemic adjuvant therapies, the mortality rate of OSCC has shown little improvement over the last two decades and overall five-year survival rate remains less than 50% [2]. The high mortality from OSCC is attributed to the presence of cervical lymph node metastasis, and diagnostic delay seems to be responsible for the poor prognosis of patients with oral cancer. Thus, the identification of molecular markers for early detection and effective treatment of OSCC is valuable and necessary.

In the present study, we focused on the enzyme Nicotinamide N-Methyltransferase (NNMT, EC 2.1.1.1.), which catalyses the N-methylation of nicotinamide, pyridines and other structural analogs, playing an important role in the biotransformation and detoxification of many xenobiotics [3], [4]. Since NNMT is reported to be overexpressed in a wide variety of tumors, we investigated whether it contributes to the carcinogenesis of OSCC *in vitro* and *in vivo*. In our previous works, we analysed NNMT expression in renal cell carcinoma (RCC) [5], OSCC [6]–[8], urothelial carcinoma (UC) of the bladder [9] and in non-small cell lung cancer (NSCLC) [10]. An increased NNMT expression was found in 100% of clear cell renal cell carcinomas, and NNMT expression levels inversely correlated with tumor size, indicating a possible role of the enzyme in tumor growth [5]. In oral squamous cell carcinoma NNMT up-regulation inversely correlated with pT, lymph node metastasis and pathological staging. Recent NNMT immunohistochemical investigations in OSCC lesions showed that NNMT overexpression is linked to tumor differentiation. Our data support the hypothesis that the enzyme plays a role in tumor expansion and may have a potential as prognostic marker for OSCC [6], [7]. Moreover, downregulation of NNMT led to decreased KB cancer cell growth, suggesting the possibility of NNMT as a therapeutic target for the treatment of cancer [11]. In a recent study, a marked increase in enzyme activity in oral cancer and an up-regulation of salivary NNMT have been shown. In addition, we reported an overexpression of tissue and urinary NNMT in bladder cancer, indicating that NNMT could represent a potential biomarker for early and non-invasive diagnosis of these malignancies [8], [9]. Moreover, we recently demonstrated that NNMT mRNA and protein levels as well as NNMT activity were increased in NSCLC samples compared with both tumor-adjacent and surrounding tissue. Interestingly, both tumor-adjacent and surrounding tissue samples of unfavourable cases (N+) display higher activity levels than those of favourable NSCLCs (N0), suggesting that normal-looking tissue of unfavourable cases seems to change toward cancer [10].

In this report, in order to explore the function of NNMT in cancer cell metabolism, we examined NNMT expression in human oral cancer cell lines by Real-Time PCR, Western blot and catalytic activity assay, and we evaluated the effect of shRNA-mediated knockdown of NNMT on cell proliferation and carcinogenesis *in vitro* and *in vivo*. NNMT was detected in all cancer cell lines tested, showing a very high expression level in PE/CA-PJ15 cells. ShRNA vectors targeted against NNMT efficiently suppressed PE/CA-PJ15 gene expression, leading to a significant decrease in cell proliferation. Reduced capacity of NNMT silenced cells to form colonies in soft agar confirmed cell growth inhibition. In addition, in nude mice NNMT downregulation induced a drastic reduction in tumor volume, suggesting the involvement of the enzyme in cancer development.

## Materials and Methods

### Cell lines and reagents

Human oral cancer cell lines (PE/CA-PJ15, PE/CA-PJ34, PE/CA-PJ41, PE/CA-PJ46, PE/CA-PJ49, HSC-2, HSC-3), purchased from the American type Culture Collection (ATCC, Rockville, MD, USA), were maintained in DMEM/F12 (PE/CA-PJ15, PE/CA-PJ34, PE/CA-PJ41, PE/CA-PJ46, PE/CA-PJ49) or RPMI 1640 (HSC-2, HSC-3) media supplemented with 10% fetal bovine serum, 100 U/ml of penicillin, 100 µg/ml of streptomycin at 37°C in a humidified 5% CO_2_ incubator.

### Animals

Six- to eight-week-old male and female athymic BALB/c nude (nu/nu) mice (average weight 30 g) were selected for this study. Mice (Harlan Laboratories, Udine, Italy) were housed in plastic cages and fed with food pellets and water *ad libitum*. The animals were maintained at constant temperature (20±1°C) and humidity (50±5%) on a 12-h light/12-h dark cycle.

The procedure and facilities complied with ethical standards and followed the requirements of Commission Directive 86/609/EEC concerning the protection of animals used for experimental and other scientific purposes. Italian legislation is defined in D.L. No. 116 of 27 January 1992. The experimental protocols were also approved by the Institutional Animal Care Committee of the Ministry of Health/Italy. All experiments were performed according to the Principles of Laboratory Animal Care. All efforts were made to minimize animal suffering and to reduce the number of animals used.

### RNA extraction

Cells were homogenized in a lysis buffer, and total RNA was extracted through the SV Total RNA Isolation System (Promega, Madison, WI, USA), according to the manufacturer's protocol. The quantity and quality of RNA were assessed spectrophotometrically at 260 nm and 280 nm, and confirmed by electrophoresis on denaturated 1% agarose gel. Total RNA (2 µg) was reverse transcribed in a total volume of 25 µl for 60 minutes at 42°C with M-MLV Reverse Transcriptase (Promega, Madison, WI, USA) using oligo(deoxythymidine)_18_ primers.

### Real-Time quantitative PCR

To examine NNMT mRNA expression quantitatively, a Real-Time PCR assay was performed using a CFX96 Real-Time PCR Detection System (Bio-Rad Laboratories, Hercules, CA, USA). cDNA, generated as described above, was used as template. To avoid false-positive results due to amplification of contaminating genomic DNA in the cDNA preparation, all primers were selected to flank an intron, and PCR efficiency was tested for both primer pairs and found to be close to 1. The following primers were used, 5′-GAATCAGGCTTCACCTCCAA-3′ (forward) and 5′-TCACACCGTCTAGGCAGAAT-3′ (reverse) for NNMT, and 5′-TCCTTCCCTGGGCATGGAGT-3′ and 5′-AGCACTGTGTTGGCGTACAG-3′ for β-actin. Both genes were run in duplicate for 40 cycles at 94°C for 30 seconds and 58°C for 30 seconds, using SsoFast EvaGreen Supermix (Bio-Rad). All samples were tested in triplicate with the reference gene β-actin for data normalization to correct for variations in RNA quality and quantity. Direct detection of PCR products was monitored by measuring the fluorescence produced by EvaGreen dye binding to double strand DNA after every cycle. These measurements were then plotted against cycle numbers. The parameter threshold cycle (Ct) was defined as the cycle number at which the first detectable increase above the threshold in fluorescence was observed. NNMT expression for each cell line was calculated by using the ΔCt, where ΔCt = Ct (NNMT)−Ct (β-actin). A small ΔCt value represents a high NNMT expression level, while a large ΔCt value is attributable to a low expression level. Following gene silencing in PE/CA-PJ15 cells, fold changes in relative gene expression were calculated by 2^−Δ(ΔCt)^ where ΔCt = Ct (NNMT)−Ct (β-actin) and Δ(ΔCt) = ΔCt (silenced cells)−ΔCt (control cells).

### Western blot analysis

Three independent Western Blot experiments were performed to evaluate NNMT protein expression level. The cell pellets (2×10^6^ cells) were homogenized in 200 µl lysis buffer (phosphate buffered saline containing 1% Nonidet P40, 0.1% sodium dodecyl sulfate, 1 mM phenylmethylsulfonyl fluoride and 2 µg/ml aprotinin). After centrifugation at 16000× g for 10 minutes at 4°C, the supernatant containing the protein extract was collected. Protein quantification of the lysates was performed by Bradford's method [12]. Samples containing 50 µg protein were subjected to 15% sodium dodecyl sulfate-polyacrylamide gel electrophoresis and transferred to polyvinylidene fluoride membranes. After regular blocking and washing the membranes were incubated with chicken polyclonal antibody (Sigma-Aldrich, St. Luis, MO) (1∶1000 dilution) against NNMT for 1 hour, followed by incubation with horseradish peroxidase conjugated rabbit anti-chicken IgG (Sigma-Aldrich, St. Luis, MO) (1∶50000 dilution) for 1 hour. NNMT protein was visualized using enhanced SuperSignal West Femto Maximum Sensitivity chemiluminescent substrate (Pierce, Rockford, IL, USA).

### NNMT Enzyme Assay

An HPLC-based catalytic assay was performed to analyze NNMT activity. A frozen cell pellet (5×10^6^ cells) was suspended in 200 µl of cold lysis buffer (50 mM tris-HCl, pH 8.6, 2 µg/ml aprotinin, 1 mM phenylmethylsulfonyl fluoride, 1 mM dithiothreitol, 1% Nonidet P40) and ½ vol glass beads. The suspension was vortexed at maximum speed for 2 minutes and then chilled on ice for 2 minutes. The homogenate was centrifuged at 16000× g for 10 minutes at 4°C. The supernatant was kept at 4°C until assayed. The standard assay mixture contained 50 mM tris-HCl, pH 8.6, 1 mM dithiothreitol, 5 mM nicotinamide, 0.5 mM S-adenosyl-L-methionine and the appropriate amount of enzyme sample to a reach final volume of 350 µl. The reaction was started by adding the substrate S-adenosyl-L-methionine. Incubations were performed at 37°C for 30 and 60 minutes. The reaction was stopped by adding 100 µl assay mixture to 50 µl ice-cold 1.2 M HClO_4_. After 10 minutes at 0°C proteins were removed by 1 minute of centrifugation in a microfuge and 130 µl perchloric acid supernatant were then neutralized by adding 35 µl 0.8 M K_2_CO_3_. The KClO_4_ so formed was removed by centrifugation. 100 µl of the neutralized supernatant was injected into a high performance liquid chromatography system 10 Dvp-uv-vis photodiode array detector (Shimadzu, Duisburg, Germany) using a 250×4.6 mm inner diameter Supelcosil® LC-18 5 µm reversed phase column. Elution conditions were as previously described [13]. Enzyme activities were tested by measuring the amount of N^1^-methylnicotinamide produced, as determined by the peak areas of the separated compound with 1 U activity representing the formation of 1 nmol N^1^- methylnicotinamide per hour of incubation at 37°C.

### ShRNA plasmid transfection and selection

For stable transfection of PE/CA-PJ15 cells, a set of pLKO.1 vectors containing stem-loop cassettes encoding short hairpin RNA (shRNA) targeted to human NNMT (Sigma-Aldrich, St. Luis, MO) were used. PE/CA-PJ15 cells (2×10^5^) were seeded in six-well plates the day before transfection. The cells were transfected with four shRNA plasmids (2.5 µg per well) against NNMT (pLKO.1-330, 1-448, 1-164, 1-711). Control cells were treated with transfection reagent only (mock). Transfection was performed using Trans It-LT1 transfection reagent (Mirus Bio Corporation, Madison, WI), according to the manufacturer's instructions. Forty-eight hours after transfection stable transfectants started to be selected by maintaining cells in complete medium containing puromicyn (1 µg/ml) with medium changes every 48 hours. For all subsequent experiments, puromicyn resistant cells were maintained in complete selection medium. The efficiency of gene silencing was detected by Real-Time PCR and Western Blot analysis. As already described for cell lines, samples containing 50 µg protein were subjected to 15% sodium dodecyl sulfate-polyacrylamide gel electrophoresis and transferred to polyvinylidene fluoride membranes. The analyses were repeated three times.

### MTT assay

Cell proliferation was determined using a colorimetric assay with 3-(4,5-dimethylthiazol-2-yl)-2,5-diphenyl tetrazolium bromide (MTT). The MTT assay measures the conversion of MTT to insoluble formazan by dehydrogenase enzymes of the intact mitochondria of living cells. After treatment with or without the appropriate plasmid, PE/CA-PJ15 cells were seeded in 96-well plates (10^4^ cells/well). Cells were allowed to attach overnight and cell proliferation was evaluated for up to 4 days by measuring the conversion of the tetrazolium salt MTT to formazan crystals. Briefly, 10 µl of MTT reagent (5 mg/ml in PBS) was added to the cells and incubated for 4 h at 37°C. The medium was removed and 200 µl of isopropanol were added. The amount of formazan crystals formed correlates directly with the number of viable cells. The reaction product was quantified by measuring the absorbance at 595 nm using an ELISA plate reader. Experiments were repeated three times. Results were analyzed and expressed as percentage of the control (control equals 100% and corresponds to the absorbance value of each sample at time zero), and presented as mean values ± standard deviation (s.d.) of three independent experiments performed in triplicate.

### Soft-agar colony formation assay

*In vitro* tumorigenicity was determined on the basis of cell growth in a soft agar colony assay. Soft agar colony-forming assay was performed in triplicate. Cells (10^4^cells/well) were seeded in 24-well plates in DMEM medium containing 0.35% low melting point (LMP) agar overlying a 0.7% LMP agar layer. The cells were cultured at 37°C in 5% CO_2_ for 30 days. Every 7 days 500 µl of fresh medium was added to each well and visible colonies were photographed.

### Tumorigenicity assay

The effect of NNMT knockdown on the tumorigenic capacity of oral cancer cells was assessed by subcutaneous injection of transfected and mock PE/CA-PJ15 cells into BALB/c nude mice, which were maintained under pathogen-free conditions. Mice were divided into two experimental groups (10 animals for each group). Mock and pLK.O1-448 silenced cells (5×10^6^ cells/mouse) were resuspended in 200 µl PBS 1X/Matrigel mixture (BD Biosciences, Bedford, MA, USA) at a 1∶1 ratio to support initial tumor growth. Control or transfected cell suspensions were inoculated subcutaneously (s.c.) on the left and right backs of nude mice. Tumor size was measured weekly with a caliper and tumor volume was calculated using the formula volume = length×width×height. After 8 weeks, all of the mice were euthanized with excess CO_2_ and tumor formation was assessed. The masses were dissected and samples were harvested for histological examination. For histology, tumors were fixed with 4% paraformaldehyde in PBS solution, and paraffin-embedded sections were prepared following routine procedures.

### Statistical analysis

Data were analyzed using GraphPad Prism software version 6.00 for Windows (GraphPad Prism Software, San Diego, CA,USA). Average values were expressed as mean ± s.d. Statistical significance between different groups was determined by repeated-measures ANOVA test. A *p* value<0.05 was accepted as statistically significant.

## Results

### NNMT mRNA expression in OSCC

**Figure 1:**
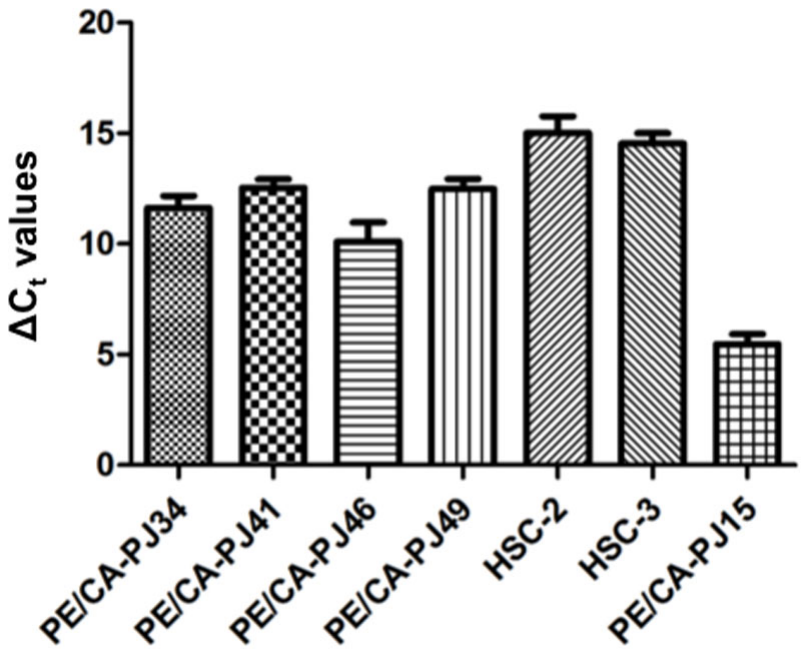
Real-Time PCR analysis of NNMT. Relative NNMT expression levels were determined in 7 human cancer cell lines. ΔCt values indicate the difference in the Ct values between NNMT and β-actin (housekeeping gene).

To quantitatively explore NNMT mRNA expression in oral cancer cell lines, total RNA isolated from cells was subjected to Real-time PCR analysis as described under Materials. As shown in Figure 1, NNMT mRNA was detected in all of the cancer cell lines tested, as its expression level was very high in PE/CA-PJ15 cells.

### Western blot analysis

**Figure 2:**
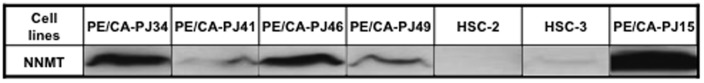
Western blot analysis of NNMT. 50 µg of protein obtained from 7 human cancer cell lines were transferred from 15% SDS-PAGE to polyvinylidene fluoride membranes. Blot was probed with anti-NNMT antibody, and detected with enhanced chemiluminescence.

To confirm the above-reported results, NNMT expression was detected at protein levels by Western Blot analysis. The analysis, performed on protein extracts obtained from OSCC cell lines, showed a single band at approximately 30 kDa, which corresponds to the known molecular mass of NNMT, with different levels of immunoreactive intensity. Weak or very weak NNMT bands were detected in four out of seven cell lines (PE/CA-PJ41, PE/CA-PJ49, HSC-2, and HSC-3) while strong NNMT bands were detected in three cell lines (PE/CA-PJ34, PE/CA-PJ46, and PE/CA-PJ15). Consistent with the results of Real Time-PCR, the strongest NNMT signal was observed in PE/CA-PJ15 cells (Figure 2).

### NNMT catalytic activity assay

**Figure 3:**
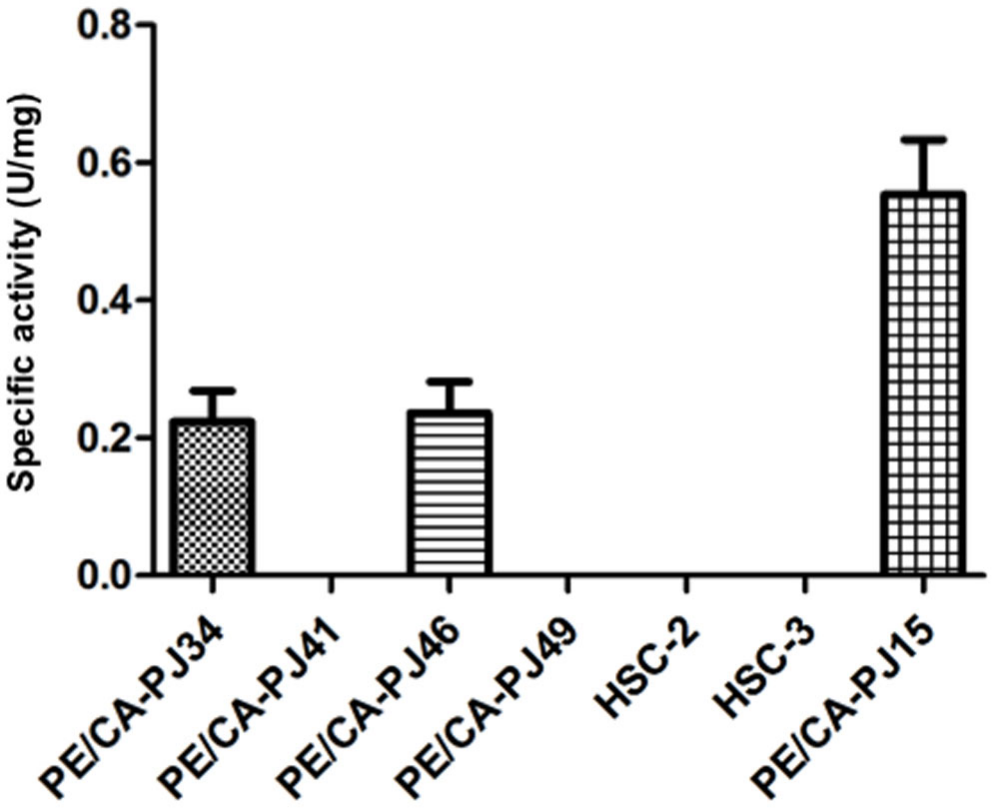
NNMT enzyme assay. NNMT specific activity in 7 human cancer cell lines was tested by measuring the amount of N^1^-methylnicotinamide produced.

An HPLC-based catalytic assay was perform to analyze NNMT activity. In keeping with the results of Real-Time PCR and Western Blot analysis, the level of NNMT specific activity, expressed in U/mg protein, was particularly high in PE/CA-PJ15 (specific activity of 0.57 U/mg) compared to the other cell lines in which activity levels were lower or undetectable (Figure 3). In most samples the activity was found to be below the detection threshold of the instrument.

### Efficiency of RNA interference

**Figure 4:**
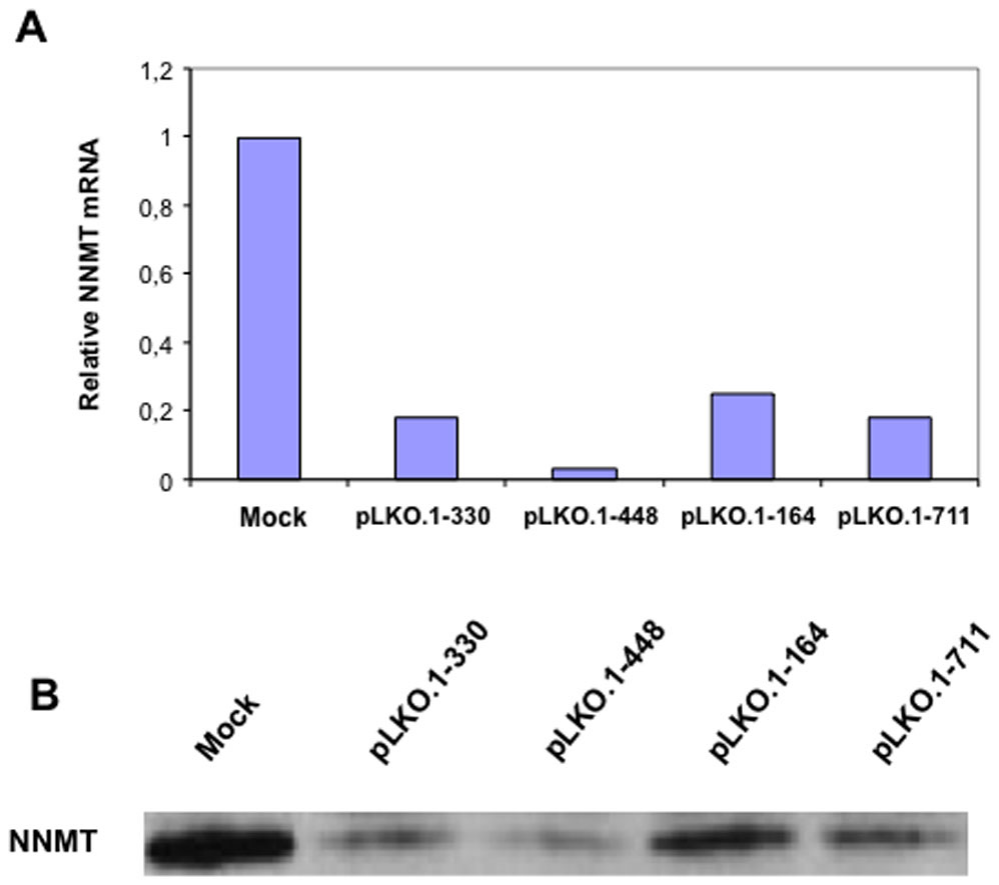
Detection of NNMT knockdown. PE/CA-PJ15 cells were treated with four shRNA plasmids against NNMT (pLKO. 1-330, 1-448, 1-164, 1-711), or with transfection reagent only (mock). (A) Real-Time PCR was used to analyze the amount of NNMT mRNA in transfected compared to mock cells. (B) Protein lysates, obtained from NNMT silenced and mock cells, were analyzed for NNMT expression by Western blot.

In order to modulate NNMT expression for functional assays, PE/CA-PJ15 cell line was stably transfected with four shRNA plasmids targeting different regions of NNMT mRNA, and control cells were treated with transfection reagent only (mock), as described in Materials and Methods. To determine the specific effects of shRNA treatment on NNMT expression, the NNMT mRNA and protein levels were analyzed by Real-Time PCR and Western blot analysis, respectively. Compared with mock, both NNMT mRNA and protein levels were significantly reduced after transfection. Mean NNMT mRNA expression was 6.25-fold lower in transfected compared to mock cells (Figure 4A). In keeping with the results of Real-time PCR, lanes loaded with equal protein amounts showed that NNMT expression was markedly decreased in transfected samples compared with that detected in mock cells (Figure 4B). Considering its remarkable silencing effect, pLKO.1-448 plasmid was used for *in vivo* experiments.

### Effect of shRNA targeting NNMT on cell proliferation

**Figure 5:**
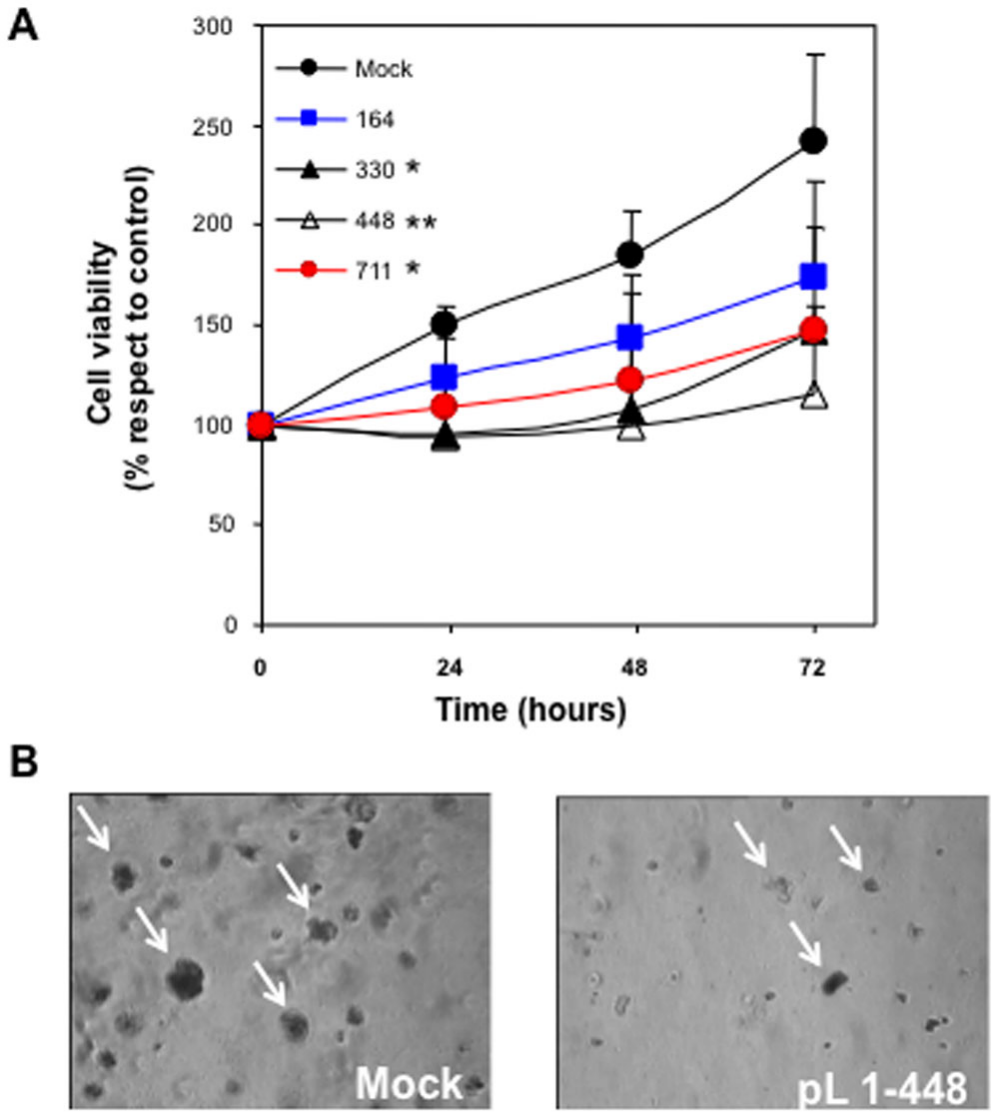
Effects of NNMT knockdown on the cell proliferation and colony formation *in vitro*. Cell proliferation was assessed by MTT analysis (A) and by soft-agar colony-forming assay (B). Cell growth and colony formation were evaluated in mock and NNMT silenced cells after 24–48–72 hours and 30 days of incubation, respectively. Three of the four plasmids were able to significantly reduce cell proliferation (*, p<0.05; **, p<0.005).

To examine the role of NNMT in tumor cell metabolism, and analyze the biological effect associated with enzyme downregulation, shRNA vectors against NNMT were introduced into PE/CA-PJ15 cells, and cell viability and colony formation were then assayed. The effect of NNMT silencing on cell proliferation was evaluated by MTT assay. As shown in Figure 5A, three of the four plasmids were able to significantly reduce cell growth (*, p<0.05; **, p<0.005). The results of MTT colorimetric assay were expressed as relative cell viability referred to control (absorbance at zero time and equal to 100%). Enzyme down-regulation resulted in reduced percentage values at all time points, the decrements being greatest with pLKO.1-448 plasmid. Cell proliferation inhibition was confirmed by soft agar colony-forming assay. The colonies formed by NNMT-transfected cells were less numerous and much smaller than those formed by mock cells (Figure 5B).

### Inhibition of *in vivo* tumor growth by NNMT downregulation

**Figure 6:**
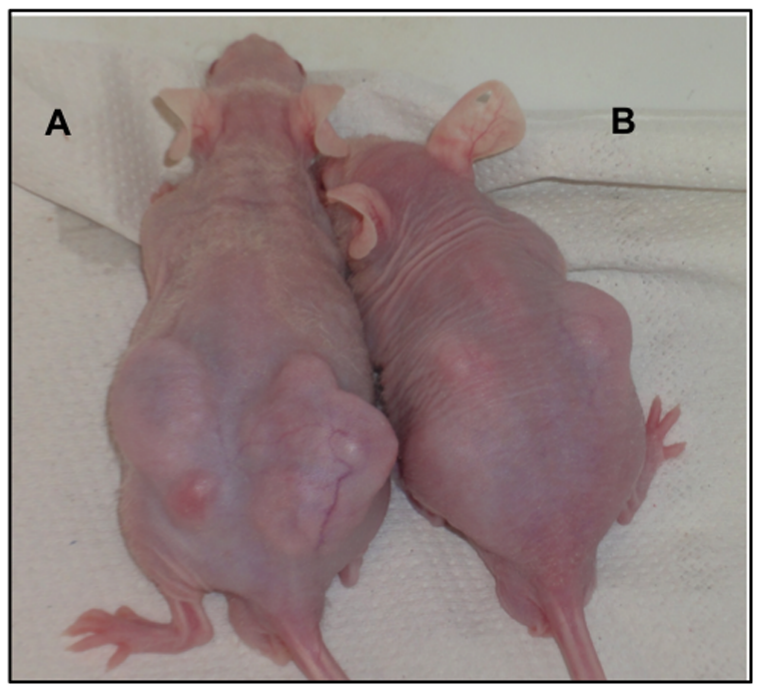
*In vivo* tumor formation of mock and silenced cells. Representative photo showing BALB/c nude mice subcutaneously injected, in the right and left flank, with mock (A) and transfected PE/CA-PJ15 cells (B), respectively.

**Figure 7:**
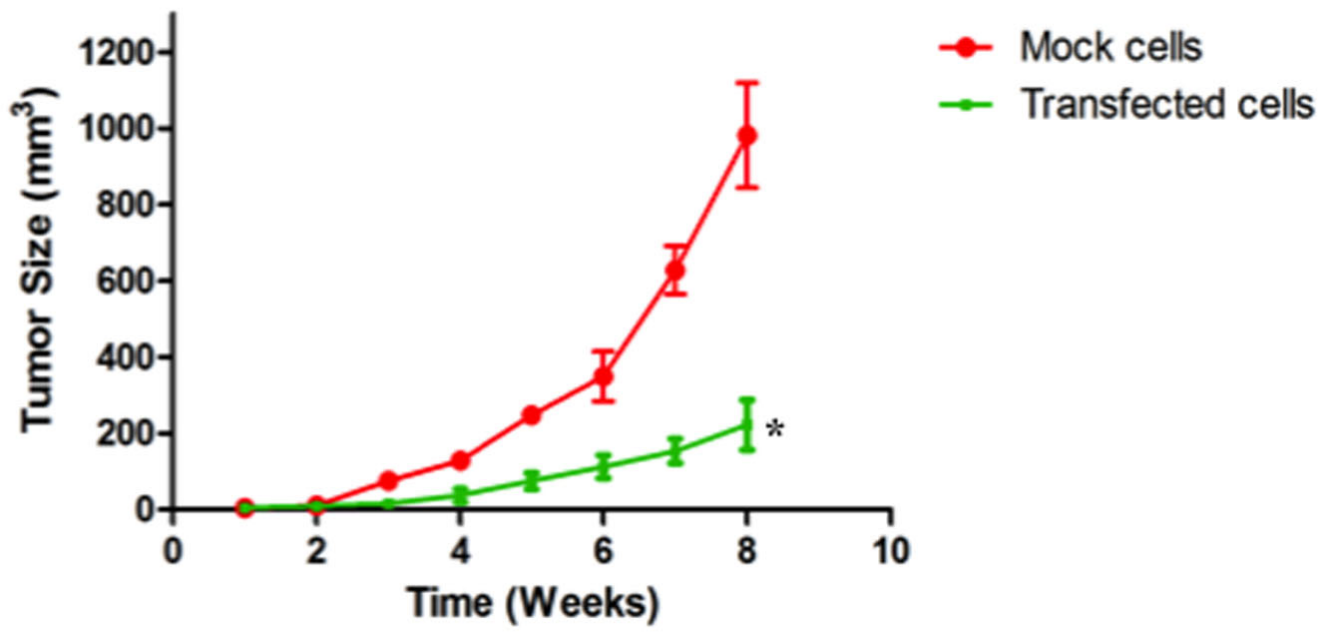
Effects of NNMT downregulation on tumor growth *in vivo*. The effect of NNMT silencing on the tumorigenic capacity was assessed by injection of mock and silenced cells into nude mice. Inoculation was performed in two groups (n = 10). Tumor size was measured weekly with caliper and the tumor volume was calculated. Data are expressed as mean of tumor volume of each group ± s.d. The differences between silenced group and control group were statistically significant (*, *p* = 0.0482).

**Figure 8:**
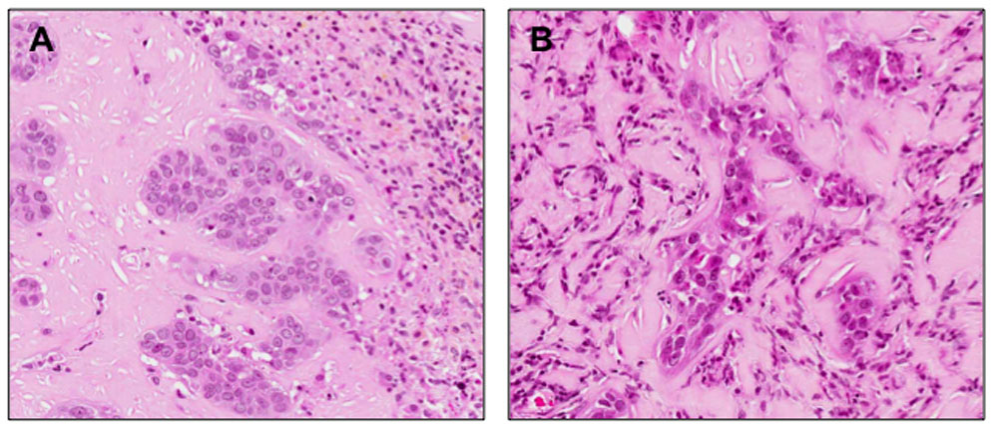
Hematoxilyn and eosin stain of xenograft tumors generated by injection of mock (A) and transfected (B) PE/CA-PJ15 cells. (**A**) Histological examination of tumor revealed nests of cohesive neoplastic epithelial cells, with connective tissue and a band of inflammatory infiltrate. (**B**) Histological examination of tumor revealed nests of neoplastic epithelial cells with ialine connective tissue and focal inflammatory infiltrate. Original magnification ×20.

To investigate whether NNMT plays a critical function in tumor formation *in vivo*, transfected and mock PE/CA-PJ15 cells (5×10^6^ cells/mouse) were subcutaneously injected on the left and right backs of athymic BALB/c nu/nu mice (10 mice/group) and tumor growth was monitored weakly. After 8 weeks, the mean size of tumors developing in mice injected with transfected PE/CA-PJ15 cells (222.3±65.5 mm^3^, n = 10) was significantly different from that in mice injected with mock PE/CA-PJ15 cells (981.7±137.5 mm^3^, n = 10). As shown in Figures 6 and 7 the growth of tumors formed by the transfected cells injection was significantly inhibited compared with tumors observed after injection of mock cells. The differences between silenced group and control group were statistically significant (*p*<0.05). At 8 weeks after inoculation all mice were killed and tumors were removed and stained with hematoxylin and eosin for histological evaluation. The morphological appearance confirmed the epithelial histogenesis of the tumor in agreement with the cell lines inoculated, excluding the formation of the primary tumor (Figure 8). These results indicated that shRNA-mediated NNMT downregulation exerted a strong growth-suppressive effect on OSCC cell lines *in vivo*.

## Discussion

Oral squamous cell carcinoma is the most common malignancy of the oral cavity. Despite latest innovations in both basic and clinical research, the overall survival rate for OSCC still remains low [14]. The high mortality associated with oral cancer is usually due to the detection of late-stage disease after the primary tumor has metastasized. The optimal management of cervical lymph node metastases is very important to improve survival, and comprehensive gene expression profiling is essential for the identification of reliable and clinically applicable markers, which allow their preoperative detection.

In the present study, we focused on the expression of NNMT, an enzyme belonging to Phase II Metabolizing Enzymes and involved in the biotransformation and detoxification of many xenobiotics. Although NNMT overespression has been reported in several kinds of tumors, the cellular effect of NNMT upregulation is still unclear. In order to explore the involvement of NNMT in oral cancer cell metabolism, we analyzed NNMT expression in seven human oral cancer cell lines and the effect of enzyme knockdown on cell growth *in vitro* and *in vivo*.

NNMT catalyzes the N-methylation of nicotinamide, pyridines and other structural analogs [3], [4]. N-methylation is one method by which drug and other xenobiotic compounds are metabolized by the liver and the enzyme NNMT is responsible for this activity which uses S-adenosyl-L-methionine as methyl donor. In the liver, where the enzyme is predominantly expressed, NNMT activity has a bimodal frequency distribution and variation in its activity might result in differences among individuals in the metabolism and therapeutic effect of drugs and in the formation of potentially toxic pyridine metabolites. Although NNMT is mainly expressed in the liver, a low NNMT expression has been also detected in the kidney, lung, skeletal muscle, placenta, heart, bladder and brain [3], [15]. Interestingly, an enhanced expression of NNMT has been reported in a number of cancers, such as glioblastoma [16], stomach adenocarcinoma [17], [18], papillary thyroid cancers [19], [20], renal [5], [21], and oral squamous cell carcinomas [6], [8], colorectal cancer [22], bladder [9], lung and pancreatic cancers [23], [24]. Moreover, it has been suggested that NNMT expression can be used as a prognostic marker for tumor [7], [25]. Among non-neoplastic diseases, an overexpression of NNMT was also detected in atherosclerosis [26], chronic obstructive pulmonary disease (COPD) [27], [28], and Parkinson's disease [29].

We have been studying the expression of NNMT in several types of malignancies for many years. In fact, the metabolism of drugs, hormones, toxic chemicals, and micronutriens is an important topic in the fields of pharmacology and endocrinology, and it is often implicated in many pathophysiological processes and diseases, such as tumorigenesis and resistance to chemotherapy [30].

In our previous reports, we analysed NNMT expression in renal cell carcinoma [5], OSCC [6], [8], bladder cancer [9] and non-small cell lung cancer [10]. Our results revealed an overexpression of NNMT in ccRCC, OSCC, bladder UC and NSCLC samples compared with adjacent noncancerous tissues, suggesting that NNMT may have potential as a biomarker for this kind of malignancies. In addition, immunohistochemical analyses showed that NNMT expression inversely correlates with OSCC histological grading, suggesting that NNMT could play a role in the early stages of oral carcinogenesis [7]. Interestingly, we recently demonstrated that NNMT expression levels were significantly higher in saliva and urine samples of patients with OSCC and bladder cancer, respectively, compared to controls. These results seem to indicate that salivary and urinary NNMT expression levels determination could be used for early and non-invasive detection of these types of cancers [8], [9].

Although abnormal NNMT expression has been identified in several types of tumors, the biological significance of alterations in NNMT activity in pathological conditions remains poorly understood, and the role of NNMT in cancer cell metabolism is still unclear. The importance of NNMT in cell proliferation and migration has recently been underlined in human bladder cancer cells, where NNMT expression was suggested to be necessary for cell growth and migration [31]. A study performed by Kim et al. on COPD showed that upregulation of NNMT in cultured skeletal myoblasts improved cell migration and proliferation and protected them against oxidant-derived injury and death [32]. Moreover, increase in NNMT expression and treatment with N^1^-methylnicotinamide significantly decreased cell death in a human neuroblastoma-derived cell line, suggesting that NNMT expression is involved in maintaining cell viability [33]. A recent investigation showed that NNMT is strongly expressed in the invasive ccRCC cell lines, and NNMT knockdown efficiently repressed metastasis formation and tumor growth of these cells *in vivo* [34]. In our previous study, we demonstrated that NNMT downregulation resulted in significantly reduced KB cancer cell proliferation, supporting the hypothesis that the enzyme may play a role in tumor growth [11].

N-methylation has been proposed as a metabolic pathway for nicotinamide excretion. Nicotinamide intracellular levels must be carefully controlled and NNMT could participate in such regulation, modulating its excretion after N-methylation. In this regard, NNMT overexpression may affect all fundamental events in which nicotinamide appears to be involved. Nicotinamide is normally present in cells and is part of the coenzyme β-nicotinamide adenine dinucleotide (NAD) molecule that influences energy cycling and cellular metabolism. Furthermore, nicotinamide has been recently proposed as a strong inhibitor of enzymes such as histone deacetylases (sirtuins) involved in stress response, regulation of apoptosis, and transcriptional silencing, and poly(adenosine diphosphate ribose) polymerase (PARP), which is fundamental for DNA damage response [35]. The lack of PARP inhibition exerted by nicotinamide, which is depleted by NNMT activity, could be the main cause for the elevated radiation resistance of a bladder cancer cell line [36], and a tumorigenic mesenchymal cancer stem cell clone [37], in which NNMT was found to be overexpressed. In addition, a recent work showed that treatment of chronic lymphocytic leukemia cells with nicotinamide blocks the deacetylating activity of endogenous SIRT1 leading to an inhibition of proliferation and to the activation of apoptosis. Moreover, the exposure to nicotinamide enhances the effect of etoposide, a DNA- damaging agent that operates through a p53-mediated apoptotic mechanism [38]. In this regard, NNMT activity, lowering nicotinamide intracellular levels, could play an important role in both tumor cell growth and chemoresistance. Increased NNMT enzyme activity in tumor cell may also affect the metabolism of antineoplastic compounds. In fact NNMT shows a low substrate specificity and its methyltransferase activity could alter the efficacy and/or adverse effect profile of standard doses of chemotherapeutic drugs.

As already mentioned, NNMT catalyzes the formation of N^1^-methylnicotinamide (MNA) that is mostly excreted into urine and partly converted via catalysis by aldehyde oxidase to N^1^-methyl-2-pyridone-5-carboxiamide and N^1^-methyl-4-pyridone-5-carboxiamide, which are also excreted into urine. Therefore, NNMT activity may also be involved in regulating the biological activity of endogenous MNA. It has recently become apparent that this compound possesses anti-thrombotic [39], vasoprotective [40], gastroprotective [41] and anti-inflammatory [42] properties. A recent work reports the neuroprotective function of N^1^-methylnicotinamide, since it reduces the toxicity of the CxI inhibitors MPP^+^ and rotenone in a human neuroblastoma-derived cell line [33]. It could be interesting to speculate whether MNA exerts a cytoprotective effect against antineoplastic compounds in cancer cells. In this regard, the significant NNMT overexpression observed in tumors, leading to elevated intracellular levels of N^1^-methylnicotinamide, could confer an adaptive advantage to cancer cells.

To our knowledge the mechanism of regulation of NNMT activity is poorly understood. Recently, NNMT promoter has been cloned and studied in papillary thyroid cancer cell lines, in which it was shown to be activated by hepatocyte nuclear factor beta (HNF-1β). HNF-1β seems to be involved in the activation of NNMT transcription, binding to specific sites in the basal promoter region [43]. Moreover, in BHP 18–21 papillary thyroid cancer cells, the histone deacetylase inhibitor depsipeptide reduced NNMT expression through HNF-1β downregulation [44]. NNMT promoter activity has also been correlated to the activation of signal transducer and activator of transcription 3 (STAT3) in colorectal tumor tissues and Hep G2 liver cancer cells stimulated with interleukin (IL)-6 [45].

Despite advances in understanding molecular mechanisms of human malignancies, the development of therapeutic approaches for the clinical treatment of human cancers remains a major challenge. In the current study, in order to investigate the potential use of NNMT as therapeutic target for human oral cancers, we employed a RNA interference technique to reduce NNMT expression in oral cancer cells and analyzed their phenotypic changes. ShRNA-directed against NNMT expressing vectors were stably transfected into PE/CA-PJ15 cells and efficiently suppressed gene expression, at both the mRNA and protein levels. NNMT downregulation led to a significant inhibition of *in vitro* proliferation and colony formation as well as decreased tumorigenic activity of OSCC cells *in vivo*. In fact, in athymic mice, NNMT silencing induced a marked reduction in tumor volume, suggesting the involvement of the enzyme in cancer development.

Although further studies are necessary to characterize the mechanism underlying NNMT expression in OSCC cell lines and clarify the role of the enzyme in cancer cell metabolism, the present study demonstrates that NNMT downregulation reduces cell proliferation as well as tumorigenic capacity of OSCC cells *in vivo*, suggesting that NNMT has not only an impact on cell growth, as previously demostrated, but also on cell tumorigenicity.

## Conclusion

The results obtained from silencing and *in vivo* experiments seem to suggest that NNMT is an important molecule involved in tumor cell proliferation and a potential target for counteracting tumor growth. Overall, this evidence indicates that NNMT may provide an excellent molecular target for oral cancer therapy. RNA interference-directed knockdown of NNMT in cancer cells might represent a convenient and novel tool for studying the biological role of NNMT and raises the potential of its application for oral cancer therapy.
